# Arid4a Suppresses Breast Tumor Metastasis by Enhancing MTSS1 Expression via mRNA Stability

**DOI:** 10.1002/cam4.70732

**Published:** 2025-03-11

**Authors:** Pengfei Wan, Dandan Zhang, Xueting Liu, Wenbao Lu

**Affiliations:** ^1^ Department of Geriatric Medicine Qilu Hospital of Shandong University Jinan Shandong China; ^2^ Institute of Microcirculation Chinese Academy of Medical Sciences & Peking Union Medical College Beijing China

**Keywords:** Arid4a, breast cancer, metastasis, mRNA stability, MTSS1

## Abstract

**Background:**

Tumor metastasis is one of the main causes of death in cancer patients; however, the mechanism controlling metastasis is unclear. The posttranscriptional regulation of metastasis‐related genes mediated by AT‐rich interactive domain‐containing protein 4A (Arid4a), an RNA‐binding protein (RBP), has not been elucidated.

**Methods:**

Bioinformatic analysis, qRT–PCR, immunohistochemistry, and immunoblotting were employed to determine the expression of Arid4a in breast tumor tissues and its association with the survival of cancer patients. In vitro and in vivo cellular experiments were used to assess the function of Arid4a in breast tumor metastasis. PCR array, RNA immunoprecipitation (RIP), luciferase, mRNA stability, RIP‐ChIP, and EMSA were conducted to elucidate the potential mechanism of Arid4a.

**Results:**

Reduced expression of Arid4a in breast tumor samples was detected via bioinformatic analyses and experimental methods. Low Arid4a expression was significantly correlated with poor prognosis in breast cancer patients. Gain‐of‐function and silencing experiments confirmed the inhibitory effect of Arid4a on tumor metastasis in vitro and in vivo. Mechanistically, Arid4a preferentially stabilizes metastasis–suppressing transcripts, including *metastasis suppressor 1* (*MTSS1*), *tissue inhibitor of metalloproteinase 2* (*TIMP2*), *retinoblastoma 1* (*Rb1*), and *phosphatase and tensin homolog* (*PTEN*), through binding to a conserved structural RNA element localized in the 3′ untranslated region (3′UTR). The Arid domain of Arid4a is required for its mRNA stabilization and metastasis inhibition. Notably, the expression of Arid4a and metastasis‐suppressing genes was positively correlated in human breast tumor tissues.

**Conclusions:**

Arid4a was confirmed to suppress breast tumor metastasis progression by stabilizing the transcripts of tumor metastasis–suppressing genes, suggesting that Arid4a might be a potential therapeutic target for breast cancer treatment.

## Graphical abstract

Here, we identified the low expression pattern of RNA‐binding protein Arid4a in human breast cancers and its potential role in modulating tumor metastasis‐related gene expression and tumor progression. Mechanistically, Arid4a could upregulate the expression of metastasis‐suppressing genes by stabilizing their mRNAs via binding to the stem–loop structure localized in 3′ UTR.

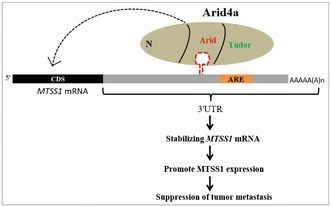

## 1 Introduction

Breast cancer is the most common cancer in women worldwide. Organ failure triggered by tumor metastasis is the main cause of patient death. Therefore, restricting tumor metastasis has become a potential strategy to control cancer progression and death [1]. The abnormal expression of metastasis‐related genes within tumor cells, such as the metastasis‐promoting genes *V‐Ki‐ras2 Kirsten rat sarcoma viral oncogene homolog* (*KRAS*) [2], *hepatocyte growth factor* (*HGF*) [3], *met proto‐oncogene* (*MET*) [4], *methionyl aminopeptidase 2* (*METAP2*) [5], and the metastasis‐suppressing genes *metastasis suppressor 1* (*MTSS1*) [6], *tissue inhibitor of metalloproteinase 2* (*TIMP2*) [7], *retinoblastoma 1* (*Rb1*) [8], and *phosphatase and tensin homolog* (*PTEN*) [9], is key for driving or limiting tumor cell metastasis. In general, tumor metastasis is driven by either the relatively increased expression of metastasis‐promoting genes or the decreased expression of metastasis‐suppressing genes [10]. Despite dramatic advances in understanding the mechanisms for regulation of metastasis‐related gene expression, the posttranscriptional effects of RBPs on these genes are still unclear.

The RNA‐binding protein Arid4a, also known as retinoblastoma‐binding protein 1 (RBBP1), belongs to the ARID (AT‐rich interaction domain) protein family [11]. Initially, Arid4a was reported to be important in epigenetic regulation and for controlling gene expression [12]. Further studies revealed that Arid4a is involved in regulating cell proliferation [13] and cell cycle progression [14] in various cell types. Structural research has demonstrated that Arid4a has several domains, including the PWWP (Pro‐Trp‐Trp‐Pro) domain [15], chromobarrel domain [16, 17], ARID domain [15], and Tudor domain [18], and can bind to DNA through its Tudor domain. Arid4a is closely associated with the pathogenic process of human diseases, including male reproductive ability [19] and cancer. Arid4a expression is suppressed in human liver cancer tissues [20] and is related to the prognosis of human breast cancer patienrs [21]. Arid4a has also been identified as a potential tumor suppressor of leukemia [22] and prostate cancer [23]. Notably, Arid4a has been reported to participate in regulating the metastasis process of multiple cancers, including breast cancer [24], thyroid cancer [25] and lung cancer [26]. These studies suggest a potential correlation between Arid4a and tumor metastasis. However, the mechanism by which Arid4a affects human breast tumor cell metastasis is still unclear.

Here, we revealed that Arid4a selectively upregulated the expression of metastasis‐suppressing genes, thereby inhibiting breast tumor metastasis. Arid4a expression is decreased in human breast tumors and several commonly used cell lines and is strongly related to poor outcomes in breast cancer patients. Exogenous expression of Arid4a effectively inhibited breast tumor cell proliferation, migration, and invasion, whereas silencing Arid4a expression promoted breast tumor metastasis. Moreover, in vivo administration of Arid4a‐expressing adenovirus inhibited breast tumor progression. Notably, the expression of metastasis‐suppressing genes, including *MTSS1*, *TIMP2*, *Rb1*, and *PTEN*, was increased according to the expression of Arid4a in breast tumor tissues. Overall, our results reveal that Arid4a inhibits breast tumor metastasis by increasing the expression of metastasis‐suppressing genes through stabilizing mRNAs, which provides a potential target for breast cancer treatment.

## 2 Materials and Methods

### 2.1 Cell Lines

Human breast cancer cell lines (MDA‐MB‐231, MDA‐MB‐468, MCF7 and T47D) and human normal mammary epithelial cell lines (MCF‐10A and MCF‐12A) were purchased from the American Type Culture Collection (ATCC). MDA‐MB‐231, MDA‐MB‐468, and MCF7 cells were cultured in Dulbecco's Modified Eagle's Medium (DMEM) supplemented with 10% FBS and 1% penicillin/streptomycin. T47D cells were cultured in Roswell Park Memorial Institute (RPMI) 1640 medium supplemented with 10% FBS plus 1% penicillin/streptomycin. MCF‐10A and MCF‐12A were cultured in DMEM containing 5% donor horse serum, EGF (20 ng/mL), hydrocortisone (0.5 mg/mL), human recombinant insulin (10 mg/mL), and 1% penicillin/streptomycin. HEK293T and HEK293 cells were purchased from the National Infrastructure of Cell Line Resource (Beijing, China) and cultured in DMEM supplemented with 10% FBS and 1% penicillin/streptomycin.

### 2.2 Antibodies and Reagents

For all the antibodies, reagents, and plasmids used in this study, see Data S1.

### 2.3 PCR Array

A QIAGEN Human Tumor Metastasis PCR Array Kit (Cat. No. 330231, PAHS‐028ZA) was used to measure the expression of metastasis‐related genes in Arid4a‐overexpressing breast tumor cells and empty vector control cells. The relative mRNA expression of each gene was calculated according to the 2^−∆∆Ct^ method, with GAPDH as an internal reference. The PCR array results are shown in Table S1.

### 2.4 Wound Healing Assay

Tumor cells overexpressing Arid4a or silencing Arid4a were grown to confluence in 35 mm dishes, and the cell layer was then scratched with 200 μL pipette tips. The scraped cells were washed with PBS and further cultured for 24 h. The scratch confluence of the cell layer was imaged with a microscope (Zeiss, Germany). ImageJ software was used to measure the open area of the wound.

### 2.5 Transwell Cell Invasion Assay

The chambers of 24‐well Transwell plates (Cat. 3422, 8.0 μm pore, Corning) were first precoated with Matrigel (BD Biosciences). Then, Arid4a‐overexpressing breast tumor cells were seeded in the upper chamber and cultured for 24 h. The chambers were fixed with 4% paraformaldehyde and dyed with 0.1% crystal violet. A cotton swab was used to remove the remaining cells from the inner side of the chambers. The chambers were inverted and imaged to visualize the invading cells (under the surface of the membrane). The invaded cells were counted and normalized to the control.

### 2.6 qRT‐PCR

One milliliter of TriZol reagent was added to each sample to extract the total RNA from the cells or tissues. Then, the RNA was reverse transcribed to cDNA using a Transcriptor First Strand cDNA Synthesis Kit (Roche). qPCR was performed using SYBR Green Fast Master Mix (Roche). GAPDH was used as an internal control for normalization of gene expression. The primers are listed in Table S2.

### 2.7 mRNA Stability

New mRNA synthesis in target cells was blocked by the addition of actinomycin D (5 μg/mL) and 5,6‐dichlorobenzimidazole riboside (5 μg/mL). Total RNA was subsequently collected at different times using TriZol. The mRNA abundance in Arid4a‐overexpression or Arid4a‐knockdown breast tumor cells was analyzed using qRT‐PCR.

### 2.8 Animal Study

The laboratory animal experimental procedures were approved by the Experimental Animal Care and Ethics Committee of the Institute of Microcirculation, Chinese Academy of Medical Sciences (CAMS) and Peking Union Medical College (PUMC). Six‐week‐old female BALB/c mice were injected with 3 × 10^6^ MDA‐MB‐231/Arid4a‐KD cells or scramble control cells into the mammary gland fat pad. For adenovirus administration, 3 × 10^6^ MDA‐MB‐231 cells were first inoculated subcutaneously into nude mice. Once the tumor mass reached a diameter of approximately 5 mm, the tumors were treated with the injection of Arid4a/GFP‐expressing adenoviruses or GFP‐expressing control adenoviruses (10^10^ pfu/tumor/time; GeneChem, Shanghai). The dynamic growth of the tumors was recorded every other day, and the tumor size was measured according to the formula length × width × height (mm^3^).

### 2.9 Statistical Analysis

GraphPad Prism 9 software was used for statistical analysis. The data are presented as the mean ± SEM. All the experiments were repeated at least three times. The Shapiro–Wilk test method was used to test the normal distribution of the data. Significant differences between two groups were calculated using Student's *t*‐test. A *p* value < 0.05 was considered statistically significant.

## 3 Results

### 3.1 Arid4a Is Expressed at Relatively Low Levels in Breast Tumors and Indicates Poor Prognosis

**FIGURE 1:**
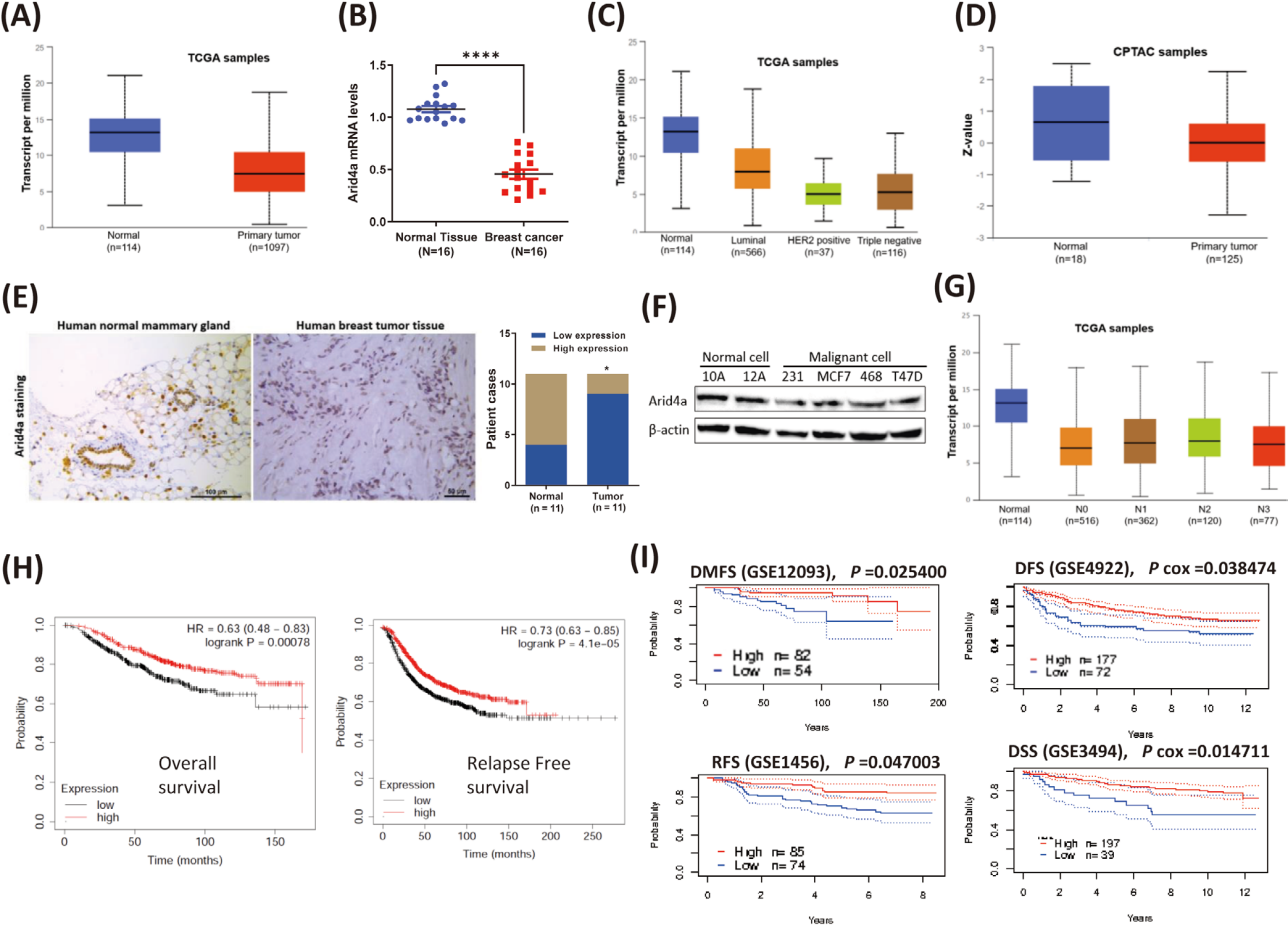
Arid4a is expressed at relatively low levels in breast tumors and indicates poor prognosis. (A) The expression of *Arid4a* between normal breast tissues and primary breast tumors was analyzed (http://ualcan.path.uab.edu/analysis‐prot.html). (B) *Arid4a* expression was measured by qPCR in human breast tumors and surrounding healthy tissues (*n* = 16). (C) Comparison of *Arid4a* expression between different subtypes of breast cancers (http://ualcan.path.uab.edu/analysis‐prot.html). (D) Arid4a protein expression in human primary breast tumors and normal tissues was analyzed (http://ualcan.path.uab.edu/analysis‐prot.html). (E) Left: Representative images of Arid4a IHC stating in normal mammary gland tissues and breast tumor tissues were shown. Scale bar, 100 μm. Right: IHC scores of Arid4a staining. (F) Arid4a protein levels were measured by immunoblotting in human normal mammary gland epithelial cell lines (MCF‐10A and MCF‐12A) and breast tumor cell lines (MDA‐MB‐231, MCF7, MDA‐MB‐468, and T47D). (G) *Arid4a* expression was analyzed based on nodal metastasis status of breast cancers (http://ualcan.path.uab.edu/analysis‐prot.html). (H) Kaplan–Meier overall survival and relapse free survival curves of breast cancer patients with low and high tumor *Arid4a* transcripts (kmplot.com). (I) Kaplan–Meier distant metastasis free survival (DMFS), disease free survival (DFS), relapse free survival, and disease specific survival (DSS) curves of breast cancer patients with low and high tumor Arid4a transcripts (PrognoScan: A new database for meta‐analysis of the prognostic value of genes. (kyutech.ac.jp)). *p < 0.05; ****p < 0.00001.

The Arid4a expression pattern in human breast tumors was first examined by surveying different TCGA databases. Relatively low expression of *Arid4a* in primary breast tumors was detected (Figure 1A, Figure S1A). *Arid4a* expression was also decreased in our breast tumor samples (Figure 1B) and different subtypes of breast cancer, including luminal, HER2+, and triple‐negative breast tumors (Figure 1C, Figure S1B). In addition, lower Arid4a protein expression was observed in primary breast tumors (Figure 1D) and various subtypes of breast tumors (Figure S1C). The reduced Arid4a expression was further confirmed by evaluating the extent of Arid4a immunohistochemistry (IHC) staining in breast tumor tissues (Figure 1E). We also found that Arid4a expression was decreased in several commonly used breast tumor cell lines (Figure 1F). Furthermore, we found that *Arid4a* expression gradually decreased with increasing breast cancer pathological stage (Figure S1D) and nodal metastasis (Figure 1G), suggesting that Arid4a expression might be involved in breast tumor metastasis. In addition, Kaplan–Meier plotting revealed that lower Arid4a expression predicted poor overall survival (OS), relapse‐free survival (RFS) (Figure 1H), distant metastasis‐free survival (DMFS), disease‐free survival (DFS), and disease‐specific survival (DSS) of patients with breast cancer (Figure 1I). Together, these data revealed that reduced Arid4a expression was closely correlated with severely poor prognosis in breast cancer patients.

### 3.2 Arid4a Impairs Breast Tumor Cell Proliferation and Metastasis In Vitro

**FIGURE 2:**
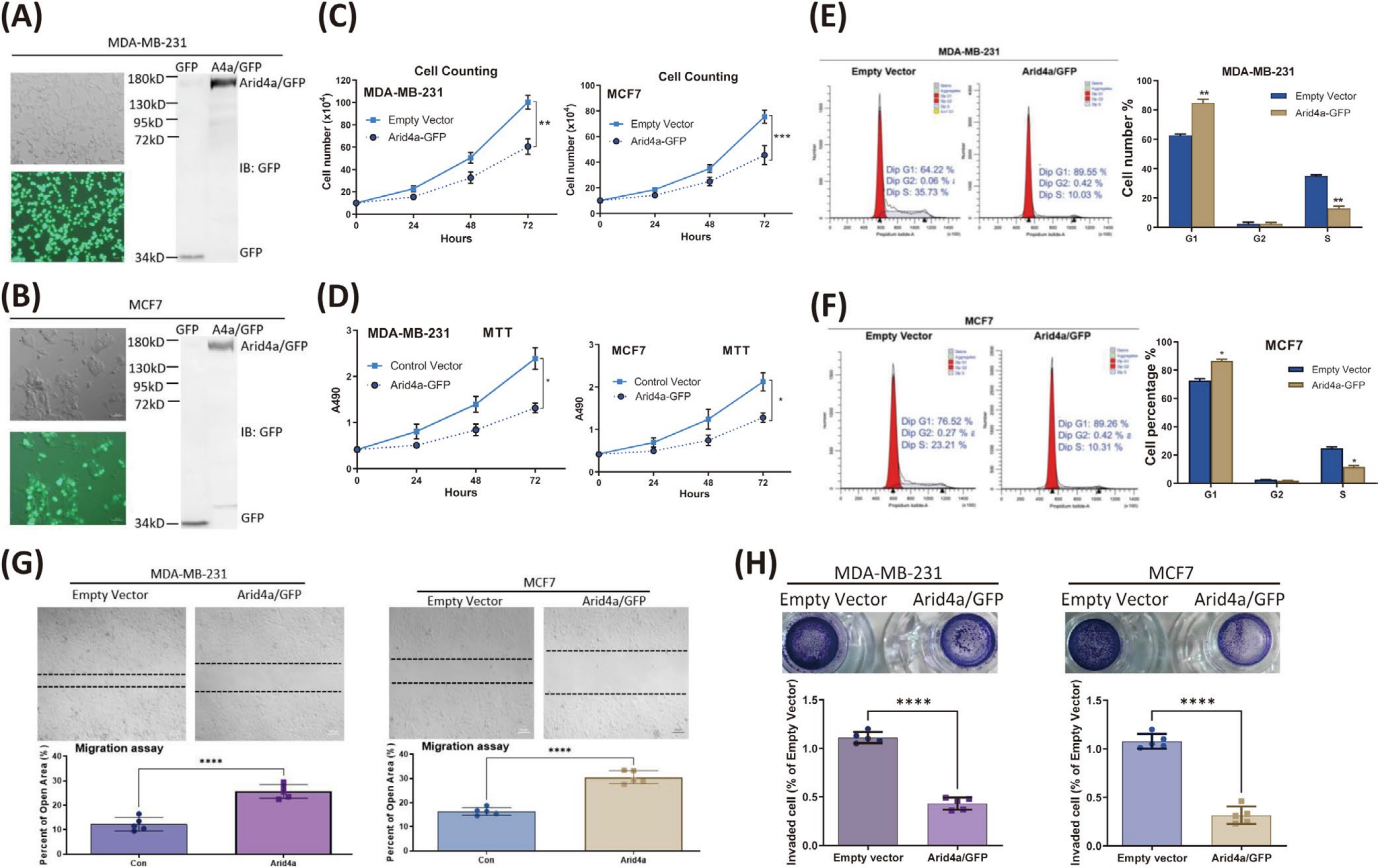
Arid4a impairs breast tumor cell proliferation and metastasis in vitro. (A, B) Arid4a/GFP fusion protein expression in MDA‐MB‐231 cells (A) and MCF7 cells (B) was identified by immunoblotting, respectively. (C) Cell counting was conducted to measure the proliferation of Arid4a‐overexpressing tumor cells. (D) MTT assay was performed to measure the cell activity of breast tumor cells overexpressing Arid4a/GFP protein. (E, F) Cell cycle was analyzed by FCM in MDA‐MB‐231 (E) and MCF7 (F) cells after Arid4a overexpression. (G) Wound healing assay was used to examine the cell migration of breast tumor cells overexpressing Arid4a/GFP protein. (H) Transwell invasion assay was conducted to measure the invasion of breast tumor cells expressing exogenous Arid4a/GFP protein. *p < 0.05; **p < 0.01; ***p < 0.0001; ****p < 0.00001.

To demonstrate the effect of Arid4a on breast tumor cell behavior, exogenous Arid4a (Arid4a‐GFP fusion protein) was expressed in MDA‐MB‐231 (Figure 2A) and MCF7 (Figure 2B) cells and confirmed by immunoblotting. Upon Arid4a overexpression, tumor cell proliferation (Figure 2C) and cell activity (Figure 2D) were substantially suppressed. We further found that Arid4a can induce cell cycle arrest in breast tumor cells. The percentage of cells in the G1 phase significantly increased after Arid4a overexpression, and the percentage of cells in the S phase decreased. We did not observe significant changes in the percentage of tumor cells in the G2 phase (Figure 2E,F). These findings suggest that Arid4a inhibits cell growth by preventing the G1/S phase transition in breast tumor cells. Since *Arid4a* expression significantly correlates with nodal metastasis in human breast cancer (Figure 1G), we next detected whether Arid4a affects the migration and invasion of breast tumor cells. Indeed, Arid4a overexpression effectively inhibited breast tumor cell migration (Figure 2G) and invasion (Figure 2H). Taken together, these results clearly indicate that Arid4a can inhibit breast tumor cell proliferation, migration, and invasion in vitro.

### 3.3 Arid4a Preferentially Promotes the Expression of Metastasis‐Suppressing Genes by Stabilizing mRNAs in Breast Tumor Cells

**FIGURE 3:**
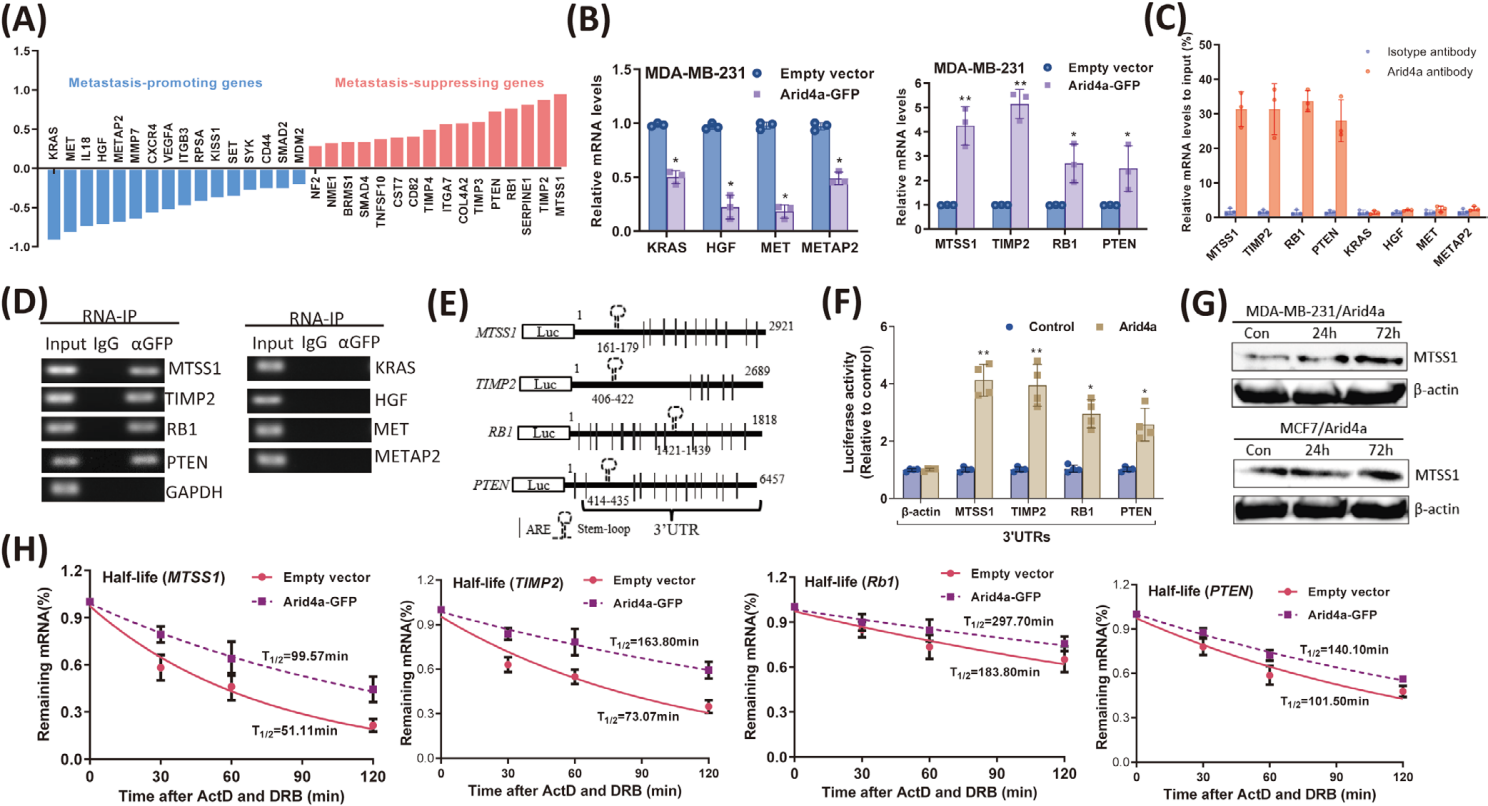
Arid4a Preferentially promotes the expression of metastasis‐suppressing genes by stabilizing mRNAs in breast tumor cells. (A) PCR Array data showing the metastasis‐promoting genes were downregulated, and the metastasis‐suppressing genes were upregulated by Arid4a in MDA‐MB‐231 cells. (B) The downregulation of indicated metastasis‐promoting mRNAs (left) and upregulation of indicated metastasis‐suppressing mRNAs (right) was confirmed by qPCR after Arid4a overexpression in MDA‐MB‐231 cells. (C) RNA‐IP with anti‐Arid4a antibody followed by qRT‐PCR detection showing that metastasis‐suppressing genes but not metastasis‐promoting genes were enriched by Arid4a. (D) RNA‐IP with anti‐GFP antibody combined with RT‐PCR detection showing that metastasis‐suppressing genes but not metastasis‐promoting genes were pulled down by Arid4a. (E) Schematic representation of the luciferase reporter constructs containing 3′ UTRs sequences of indicated metastasis‐suppressing genes. (F) The relative luciferase activities of the indicated reporters were measured by the luciferase reporter assay. (G) MTSS1 and β‐Actin protein expression were analyzed by western blotting in Arid4a overexpressed MDA‐MB‐231 and MCF7 cells, respectively. (H) Half‐lives of indicated metastasis‐suppressing transcripts were prolonged by Arid4a in MDA‐MB‐231 cells. *p < 0.05; **p < 0.01.

Given that Arid4a can inhibit the migration and invasion of breast tumor cells, we next identified the metastasis‐associated genes affected by Arid4a using the Human Tumor Metastasis RT2 Profiler PCR Array (QIAGEN). A total of 16 metastasis‐promoting genes were downregulated (< −0.2‐fold) and 16 metastasis‐suppressing genes were upregulated (> 0.2‐fold) in Arid4a‐expressing MDA‐MB‐231 cells (Figure 3A). The detailed PCR array data are summarized in Table S1. To confirm our PCR array results, the mRNA expression of four downregulated metastasis‐promoting genes (*KRAS*, *HGF*, *MET*, and *METAP2*) and four upregulated metastasis‐suppressing genes (*MTSS1*, *TIMP2*, *RB1*, and *PTEN*) was tested using real‐time PCR. All four metastasis‐promoting gene mRNAs were decreased in Arid4a‐overexpressing cells, and all the metastasis‐suppressing genes increased upon Arid4a overexpression in breast tumor cells (Figure 3A, Figure S2A,B). To determine whether Arid4a bound to these metastasis‐associated genes, RNA immunoprecipitation (RIP) followed by real‐time PCR was performed using MDA‐MB‐231 cell extracts with an anti‐Arid4a antibody. All four metastasis‐suppressing genes, but not the metastasis‐promoting genes, were enriched in the Arid4a antibody‐pulldown complex (Figure 3C). We further confirmed this by performing RIP using a specific anti‐GFP antibody and its isotype IgG following RT–PCR detection. Metastasis‐suppressing genes were detected by PCR, but metastasis‐promoting genes were not (Figure 3D). These results suggest that Arid4a selectively upregulates the expression of metastasis‐suppressing genes to inhibit breast tumor cell metastasis.

Studies have reported that RBPs bind to mRNAs through targeting the 3′ untranslated region (3′ UTR) [27]. To identify whether Arid4a also targets the 3′ UTRs of metastasis‐suppressing mRNAs, the full‐length 3′ UTRs of *MTSS1*, *TIMP2*, *RB1*, and *PTEN* were cloned and inserted downstream of the luciferase gene (Figure 3E). The luciferase activities of four reporters containing different 3′ UTRs of metastasis‐suppressing genes were increased, suggesting that Arid4a targets metastasis‐suppressing mRNAs by recognizing the 3′ UTR (Figure 3F). In addition, we observed that MTSS1 expression was increased by Arid4a over time in breast tumor cells (Figure 3G). We subsequently examined whether Arid4a increases the expression of metastasis‐suppressing genes through regulating mRNA stability. The half‐lives of metastasis‐suppressing mRNAs, including *MTSS1*, *TIMP2*, *RB1*, and *PTEN*, were prolonged by approximately 1‐fold in Arid4a‐overexpressing breast tumor cells (Figure 3H). Together, these findings indicate that Arid4a stabilizes metastasis‐suppressing mRNAs through targeting the 3′ UTR in breast tumor cells.

### 3.4 Arid4a Binds to the Conserved RNA Secondary Structure but Not the Linearized RNA Sequence in the 3' UTR of *MTSS1*

**FIGURE 4:**
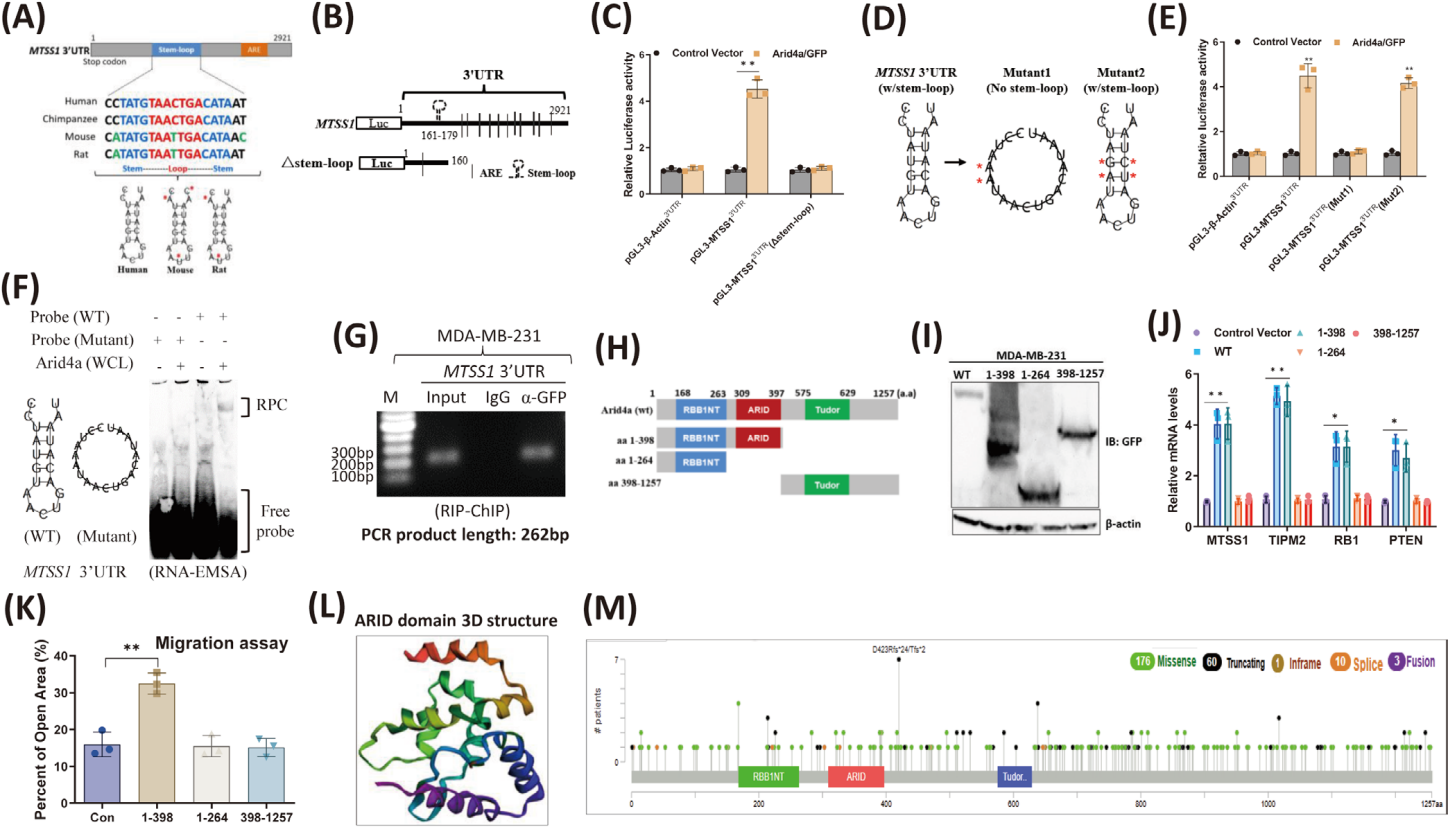
Arid4a binds to the conserved RNA secondary structure but not the linearized RNA sequence in the 3′ UTR of *MTSS1*. (A) Alignment of the conserved stem–loop sequences in *MTSS1* across different species and the diagram of stem–loop structure. (B) Luciferase reporter constructs of *MTSS1* containing truncated 3′ UTRs without the stem–loop structure. (C) Relative luciferase activities of the indicated reporters were measured by the luciferase reporter assay. (D) Mutation strategy of *MTSS1* stem–loop structure (asterisks indicate base substitution). Mutant1 losses the stem–loop structure (middle) and Mutant2 still forms a stem–loop structure (right). (E) Relative luciferase activities of the indicated reporters were determined by luciferase assay. (F) RNA‐EMSA showed that a unique RNA‐protein complex (RPC) formed using biotin‐labeled *MTSS1* stem–loop probes and whole‐cell lysates (WCL) extracted from MDA‐MB‐231/Arid4a/GFP cells. (G) RNA‐ChIP was performed with genome fragments from MDA‐MB‐231/Arid4a/GFP cells. (H) Schematic representation of the domains in Arid4a and the truncation mutants. (I) Expression of Arid4a truncations was confirmed by immunoblotting with anti‐GFP antibody. (J) Expression of indicated metastasis‐suppressing genes was detected by qRT‐PCR after overexpressed Arid4a and those mutants in MDA‐MB‐231 cells. (K) The cell migration of breast tumor cells overexpressing different truncations was detected by performing wound healing assay. (L) Structure of ARID domain was showed by the cBioPortal tool (cBioPortal for Cancer Genomics). (M) The mutation sites of Arid4a in human breast cancer were displayed according to cBioPortal (cBioPortal for Cancer Genomics). *p < 0.05. **p < 0.01.

Previous studies reported that ARID family proteins bind to mRNAs through the conserved structural RNA motif in the 3′ UTR [28]. A conserved sequence was identified when the 3′ UTR sequences of the *MTSS1* gene were aligned among different species. Although the conserved sequences varied among human, mouse, and rat species, all the consensus sequences could fold into a stem–loop secondary structure (Figure 4A). To confirm whether this stem–loop structure is critical for Arid4a‐mediated mRNA stability, a deletion mutant reporter of the *MTSS1* 3′ UTR without the stem–loop structure was constructed (Δstem–loop) (Figure 4B). Compared with that of the reporter containing the MTSS1 full‐length 3′ UTR, the luciferase activity of the Δstem–loop reporter was not increased by Arid4a (Figure 4C). To further confirm the importance of the secondary conformation for Arid4a‐mediated mRNA stability, we generated two mutant reporters of the *MTSS1* 3′ UTR. The secondary conformation of mutant 1 was completely disrupted, but mutant 2 still maintained the secondary conformation despite the substitution of nucleotides in the stem region (Figure 4D). The luciferase activity of mut 2, but not mut 1, was increased by Arid4a (Figure 4E). RNA‐EMSA further revealed that only the probe with a secondary conformation could form a unique band but not the linearized mutant probe (Figure 4F), suggesting that secondary confirmation is necessary for the interaction between Arid4a and mRNA. Furthermore, the in vivo binding of Arid4a to the stem–loop structure was confirmed by RNA immunoprecipitation‐chromatin immunoprecipitation (RIP‐ChIP) (Figure 4G). These results demonstrate that Arid4a stabilizes *MTSS1* mRNA through recognition of the RNA secondary structure in the 3′ UTR independent of the linearized nucleotide sequence.

To identify the domain of Arid4a required for *MTSS1* mRNA stability, three truncations with different domains of Arid4a were generated (Figure 4H) and confirmed by immunoblotting (Figure 4I). The mRNA expression of metastasis‐suppressing genes was effectively inhibited by the aa 1–398 mutation but not by the aa 1–264 and aa 398–1257 mutants (Figure 4J). Moreover, the migration of MDA‐MB‐231 cells was significantly inhibited by the mutant aa 1–398 but not by the other mutants (Figure 4K, Figure S3A), indicating that the ARID domain is critical for Arid4a‐induced suppression of tumor metastasis. The 3D structure of the ARID domain is shown in Figure 4L. In addition, we also examined the genetic alteration of the ARID domain in human breast cancer and reported that highly dense ‘missense’ mutations exist in this domain (Figure 4M). Moreover, ‘amplification’ and ‘mutation’ were the two primary alteration frequencies of Arid4a in breast cancer patients (Figure S3B). Overall, these findings suggest that the ARID domain of Arid4a is important for mediating breast tumor metastasis and is associated with tumor progression.

### 3.5 Knockdown of Arid4a Decreases the Stability of Metastasis‐Suppressing mRNAs and Promotes Breast Tumor Metastasis Progression

**FIGURE 5:**
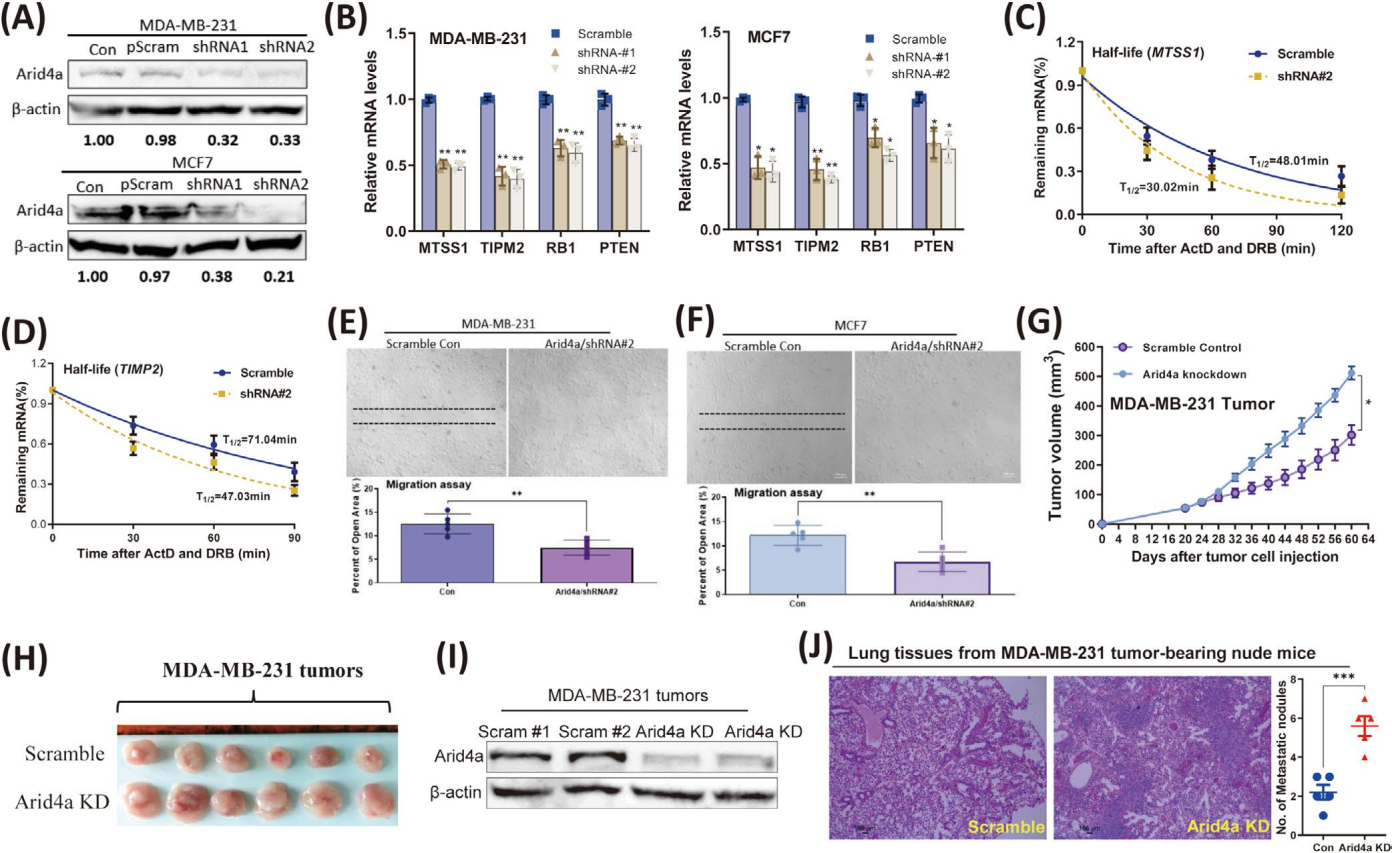
Knockdown of Arid4a decreases the stability of metastasis–suppressing mRNAs and promotes breast tumor metastasis progression. (A) Arid4a knockdown was validated by immunoblotting in MDA‐MB‐231 and MCF7 cells, respectively. (B) qRT‐PCR was used to detect the expression of indicated metastasis‐suppressing mRNAs in MDA‐MB‐231 (left) and MCF7 (right) cells after Arid4a knockdown. (C, D) Half‐lives of the indicated mRNAs were decreased upon Arid4a knockdown in MDA‐MB‐231 cells. (E, F) Cell migration of MDA‐MB‐231 cells (E) and MCF7 cells (F) was measured, respectively, after knocked down of Arid4a. (G) MDA‐MB‐231 tumor growth curves in nude mice after knocked down Arid4a. (H) Comparison of MDA‐MB‐231 tumors expressing scramble control or Arid4a/shRNAs (Arid4a KD). (I) Arid4a knockdown in MDA‐MB‐231 tumor model was confirmed by performing western blot. (J) H&E staining of lung tissue sections from nude mice bearing MDA‐MB‐231/Scramble or MDA‐MB‐231/Arid4a KD tumors. Scale bar, 100 μm. Quantification of metastatic nodules were shown in the right panel. *p < 0.05; **p < 0.01; ***p < 0.0001.

Corresponding to the overexpression results, we next examined the effects of Arid4a knockdown on breast tumor metastasis. The western blotting results confirmed the knockdown of Arid4a in breast tumor cells (Figure 5A). We first found that silencing Arid4a expression promoted breast tumor cell proliferation (Figure S4A) and activity (Figure S4B). We demonstrated that the mRNA expression of metastasis‐suppressing genes was markedly decreased by Arid4a knockdown (Figure 5B). Conversely, the mRNA levels of metastasis‐promoting genes were not significantly affected after Arid4a knockdown (Figure S4C). Moreover, the half‐lives of metastasis‐suppressing gene mRNAs were shortened upon depletion of Arid4a in MDA‐MB‐231 cells (Figure 5C,D), except for the half‐lives of metastasis‐promoting mRNAs (Figure S4D). As expected, silencing Arid4a significantly promoted the migration of breast tumor cells in vitro (Figure 5E,F). In addition, Arid4a knockdown promoted tumor growth (Figure 5G–I) and lung metastasis (Figure 5J) in vivo. Overall, these results demonstrate that a reduction in Arid4a decreased the mRNA stability of metastasis‐suppressing genes and promoted the progression of breast cancer metastasis.

### 3.6 Arid4a Suppresses Breast Tumor Progression and Is Positively Correlated With *MTTS1* Expression in Human Breast Tumors

**FIGURE 6:**
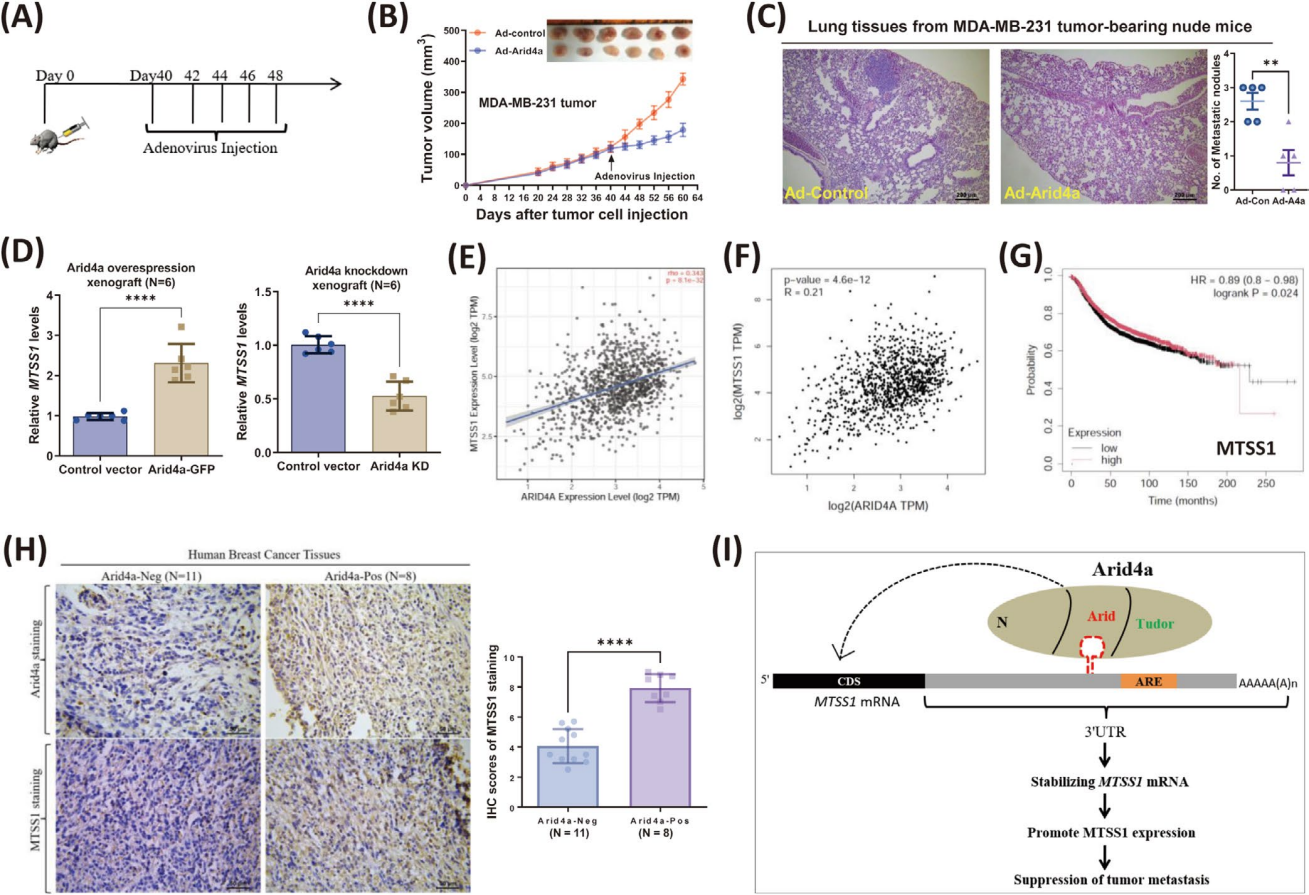
Arid4a suppresses breast tumor progression and is positively correlated with *MTTS1* expression in human breast tumors. (A) Experimental flow chart for tumor treatment with Arid4a‐expressing adenovirus in vivo. (B) Tumor images and growth curves after treatment with adenovirus. (C) H&E staining of lung sections from tumor‐bearing mice treated with control adenovirus or Arid4a‐expressing adenovirus, respectively. Scale bar, 200 μm. (D) qRT‐PCR was performed to examine the mRNA expression of *MTSS1* in Arid4a overexpression xenografts (left) and Arid4a knockdown xenografts (right). (E, F) The positive correlations between Arid4a expression and MTSS1 expression were analyzed in human breast cancer patients by TIMER2.0 (TIMER2.0 (cistrome.org)) and GEPIA2 (http://gepia2.cancer‐pku.cn/#analysis), respectively. (G) Kaplan–Meier relapse free survival curves of breast cancer patients with low and high tumor *MTSS1* transcripts. (H) Left: Representative images of IHC staining for Arid4a (up) and MTSS1 (down) in human breast cancer tissues. Scale bar, 50 μm. Right: IHC scores of MTSS1 staining in breast tumors. (I) A working model of Arid4a‐mediated metastasis‐suppressing mRNAs stability and suppression of tumor metastasis in breast cancer. **p < 0.01; ****p < 0.00001.

To test the effect of Arid4a on the progression of breast cancer, we treated established breast tumors with an Arid4a‐expressing adenovirus (Figure 6A). Compared with control adenovirus injection, treatment with Arid4a‐expressing adenovirus significantly inhibited tumor growth (Figure 6B) and lung metastasis (Figure 6C). We further found that the expression of *MTSS1*, a tumor suppressor, was decreased in human breast tumors (Figure S5A–C). Interestingly, the expression of *MTSS1* was increased by Arid4a in Arid4a‐overexpressing xenografts but decreased in Arid4a‐knockdown tumors (Figure 6D). Moreover, the expression of metastasis‐suppressing genes, including *MTSS1, TIMP2, Rb1*, and *PTEN*, gradually increased with increasing *Arid4a* expression in human breast cancer samples (Figure 6E,F, Figure S5D). Notably, lower MTSS1 expression was associated with poorer prognosis in patients with breast cancer (Figure 6G). Furthermore, our IHC results confirmed that the expression of MTSS1 is greater in human breast tumor tissues highly expressing Arid4a (Figure 6H). These findings indicate that Arid4a stabilizes MTSS1 mRNA through binding to the structural RNA in the 3′ UTR via the Arid domain, resulting in the upregulation of MTSS1 and the suppression of metastasis (Figure 6I).

## 4 Discussion

To date, the RNA‐binding protein Arida has been implicated in the metastasis progression of human cancers, including thyroid cancer, breast cancer, lung cancer, and prostate cancer [24, 25, 26]. However, how Arid4a regulates breast tumor metastasis has not been reported. In this study, we revealed that the expression of Arid4a is impaired in human breast tumor tissues and strongly correlates with patient prognosis. Arid4a overexpression effectively inhibits breast tumor growth and metastasis both in vitro and in vivo. Notably, we noted that there was a significant correlation between Arid4a expression and nodal metastasis in breast cancer, which implied that Arid4a might be a predictive biomarker of breast tumor metastasis progression.

Currently, few studies have investigated the effects of Arid4a on the behavior of breast tumor cells. We demonstrated the inhibitory roles of Arid4a in regulating the proliferation, cell cycle progression, migration, and invasion of breast tumor cells. These findings suggest that Arid4a might suppress breast tumor progression by targeting multiple signaling pathways. Arid4a functions as a ‘switch’ of tumor metastasis by mediating the balance of metastasis‐promoting and metastasis‐suppressing genes in human breast tumor cells. We speculated that the downregulation of metastasis‐promoting genes might be a secondary effect of Arid4a because Arid4a cannot directly bind to metastasis‐promoting mRNAs. Moreover, we confirmed the importance of the ARID domain for Arid4a‐mediated metastasis suppression by examining different truncations.

Among the targets of Arid4a, MTSS1, RB1, and PTEN are involved in the suppression of tumor cell metastasis, while TIMP2 is believed to be a suppressor of tumor angiogenesis [29]. Thus, Arid4a might suppress breast tumor metastasis by inhibiting the tumor angiogenesis pathway, which would require further study. The 3′ UTR is important for RBP binding to target genes [30]. We demonstrated that Arid4a could also target the 3′ UTR of metastasis‐suppressing mRNAs using a luciferase reporter assay. Besides, the half‐lives of metastasis‐suppressing mRNAs are prolonged by Arid4a, indicating for the first time that Arid4a stabilizes the metastasis‐suppressing transcripts through targeting the 3′ UTR in human breast tumor cells.

MTSS1, TIMP2, RB1, and PTEN were selected as targets of Arid4a in breast tumor cells for further study for two main reasons: (i) they are among the genes most highly regulated by Arid4a in breast tumor cells; and (ii) they are known typical metastasis inhibitory genes. Among them, MTSS1 is a potent tumor metastasis suppressor that is implicated in the metastasis suppression of various human cancers [31, 32, 33]. Here, we elucidated a novel mechanism by which Arid4a regulates MTSS1 expression. We found that a conserved sequence in the 3′ UTR of MTSS1 can fold into an RNA stem–loop structure. In addition, the secondary structure of the 3′ UTR of the MTSS1 transcript but not the AU‐rich element (ARE) is required for Arid4a‐mediated mRNA binding and stability. Our previous research revealed that several RBPs can bind to structural RNA, including Roquin1/2 [34, 35], SAMD4A (Sterile alpha motif domain‐containing protein 4A) [36], TARBP2 (*Trans*‐activation response (TAR) RNA‐binding protein 2) [37], and MCPIP1 (monocyte chemotactic protein‐induced protein 1) [38]. These findings indicate that the RNA secondary structure is crucial for RBP‐mediated gene expression, and an open question is how the different RNA‐binding domains recognize the structural RNA; further investigation is still necessary. Moreover, the positive correlation between the expression levels of Arid4a and metastasis‐suppressing genes in human breast tumors further suggests an association between Arid4a and metastasis suppression.

## 5 Conclusions

In summary, we revealed the low expression pattern of Arid4a in breast cancer and its inhibitory effect on tumor metastasis progression. Moreover, we identified the transcripts of metastasis‐suppressing genes as downstream targets of Arid4a. As an example, Arid4a was found to stabilize the mRNA of the MTSS1 gene by binding to the structural RNA in the 3′ UTR through its Arid domain. In addition, the induction of Arid4a in vivo inhibits the growth and metastasis of breast tumors. Therefore, our findings showed that Arid4a, as a metastasis suppressor, might be a promising therapeutic target for breast cancer.

## Author Contributions

**Pengfei Wan:** data curation (lead), formal analysis (lead), investigation (equal), methodology (equal), validation (equal), visualization (equal), writing – original draft (equal). **Dandan Zhang:** data curation (equal), methodology (equal). **Xueting Liu:** methodology (equal). **Wenbao Lu:** conceptualization (lead), funding acquisition (lead), project administration (lead), supervision (lead), writing – review and editing (lead).

## Ethics Statement

The study was conducted in accordance with the Declaration of Helsinki and approved by the Medical Ethics Committee of the Institute of Microcirculation, Chinese Academy of Medical Sciences & Peking Union Medical College.

## Conflicts of Interest

The authors declare no conflicts of interest.

## Supporting information


**Figure S1** Arid4a expression was reduced in human breast cancer tissues.
**Figure S2** Arid4a regulates the mRNA expression of metastasis‐related genes in breast tumor cells.
**Figure S3** Arid4a inhibits breast tumor cell migration by ARID domain.
**Figure S4** Knockdown of Arid4a increases cell growth of breast tumor cells.
**Figure S5** Arid4a expression was positively correlated with MTSS1 expression in human breast cancer tissues.

**Table S1** PCR Array analysis of human metastasis‐related genes regulated by Arid4a.

**Table S2** PCR Primers and RNA‐EMSA Probes Sequence.

Data S1

## Data Availability

All supporting data of this study are included in the manuscript and/or the Supporting Information. Any additional information or data are available upon request.
