# Supplementary material for: Arid4a Suppresses Breast Tumor Metastasis by Enhancing MTSS1 Expression via mRNA Stability
Source: Cancer Med. 2025 Mar 11;14(5):e70732. doi: 10.1002/cam4.70732 (PMC11894439; doi:10.1002/cam4.70732)
Supplement: Supplementary file 1 — Figure S1 Arid4a expression was reduced in human breast cancer tissues. Figure S2 Arid4a regulates the mRNA expression of metastasis‐related genes in breast tumor cells. Figure S3 Arid4a inhibits breast tumor cell migration by ARID domain. Figure S4 Knockdown of Arid4a increases cell growth of breast tumor cells. Figure S5 Arid4a expression was positively correlated with MTSS1 expression in human breast cancer tissues. [file CAM4-14-e70732-s004.docx]

**Figure S1. Arid4a expression was reduced in human breast cancer tissues.** (A) The expression of Arid4a gene in breast cancers was analyzed through GEPIA2 (<http://gepia2.cancer-pku.cn/#analysis>). (B) Expression of *Arid4a* in breast cancers and other human cancers was analyzed by TIMER2.0 ([timer.cistrome.org](http://timer.cistrome.org/)). (C) Protein expression of Arid4a in different subtypes of breast cancers (<http://ualcan.path.uab.edu/analysis-prot.html>). (D) Expression of *Arid4a* were analyzed by the main pathological stages of breast cancer (<http://ualcan.path.uab.edu/analysis-prot.html>).

**
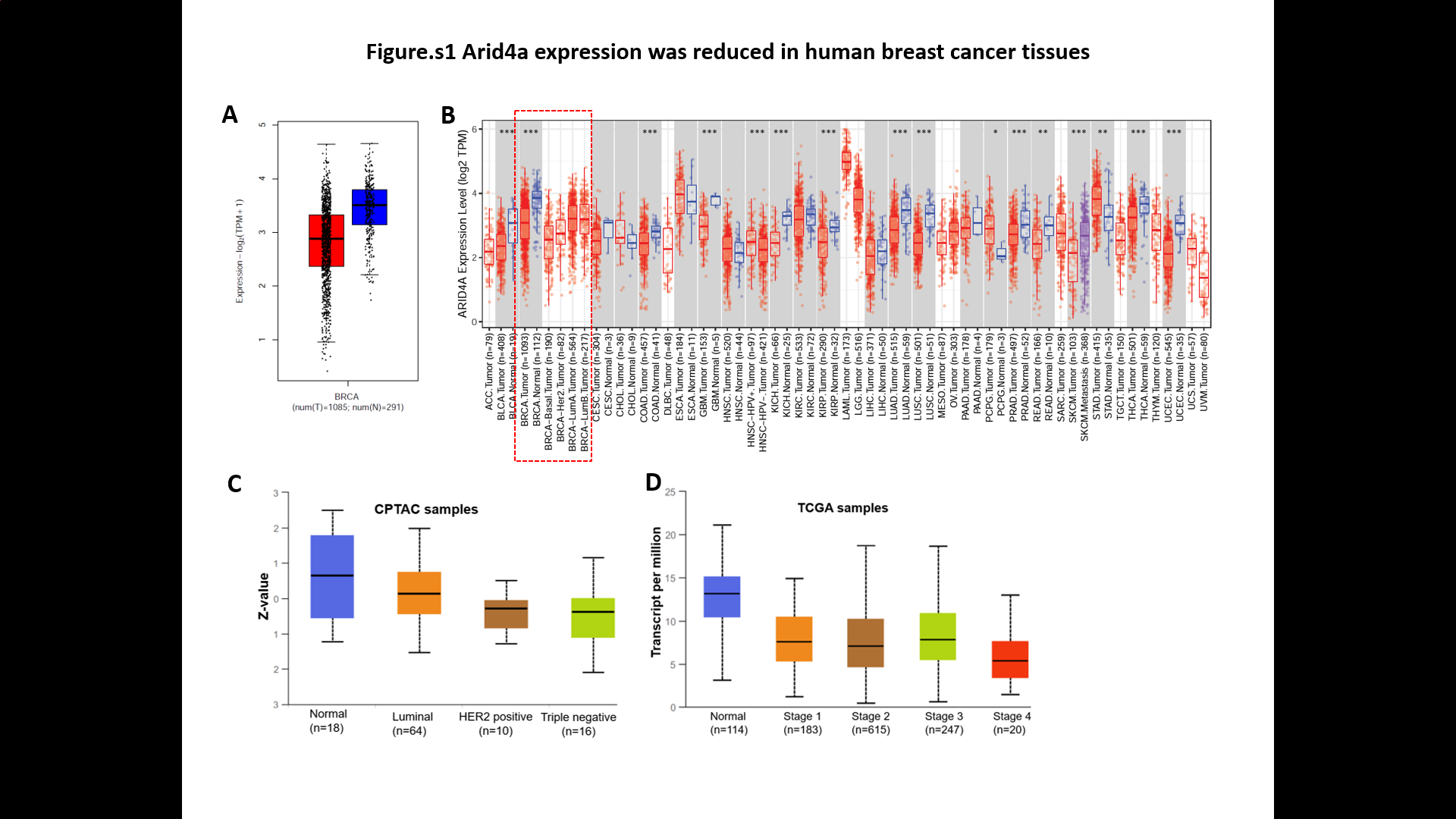
**

**Figure S2. Arid4a regulates the mRNA expression of metastasis-related genes in breast tumor cells. (**A, B) qPCR confirming the downregulation of indicated metastasis-promoting genes (A) and upregulation of indicated metastasis-suppressing gene (B) after Arid4a overexpression in MCF7 cells. **P*<0.05, ***P*<0.01.


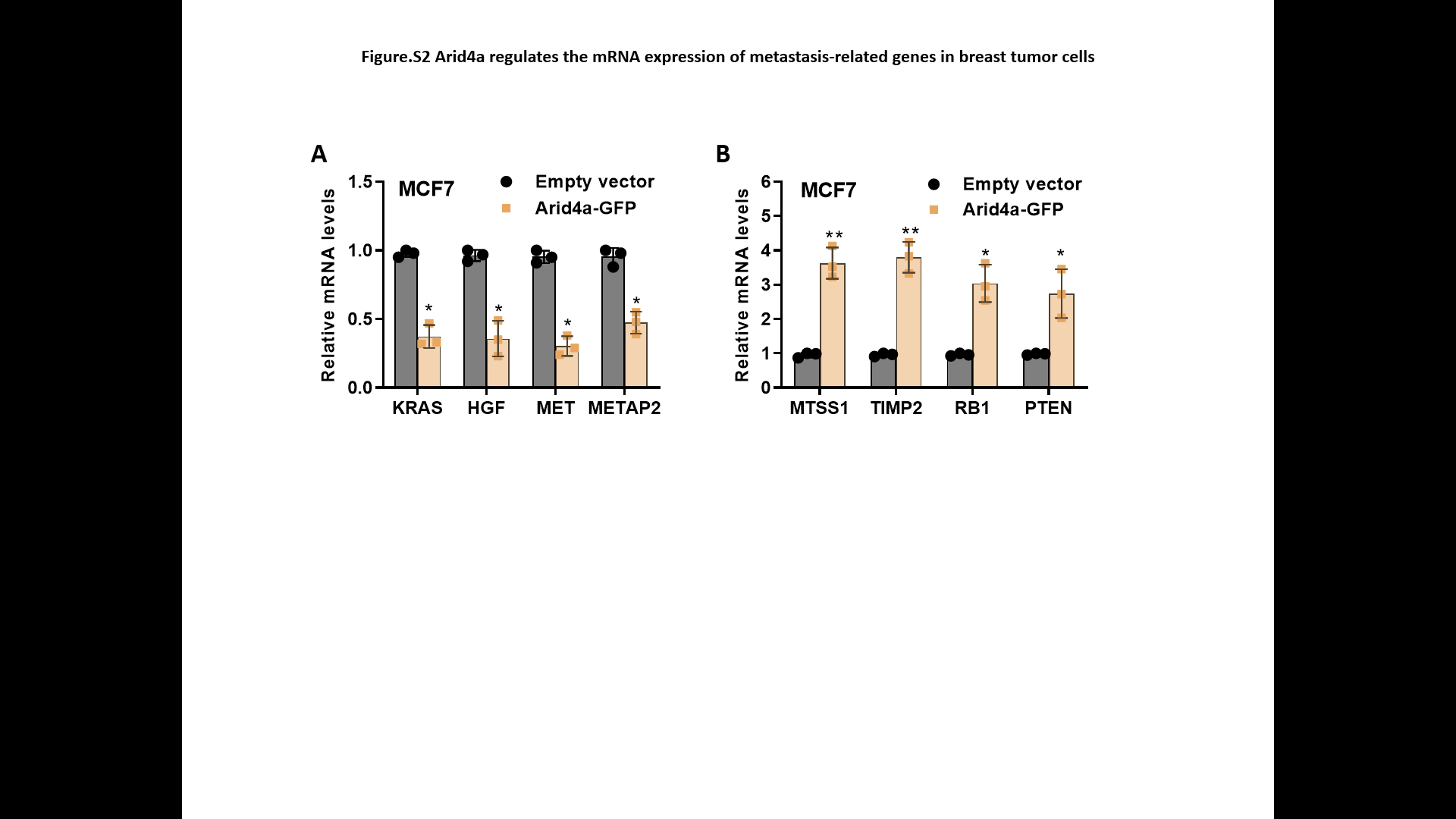


**Figure S3. Arid4a inhibits breast tumor cell migration by ARID domain. (A)** Representative photographs of migrated tumor cells after overexpression of indicated Arid4a truncations in MDA-MB-231 cells. Scale bar, 100µm. (**B)** Analysis of Arid4a alteration frequency in different human caner types according to cBioPortal dataset. The frequency of Arid4a alteration with “mutation” and “amplification” were highest in breast cancer.

**
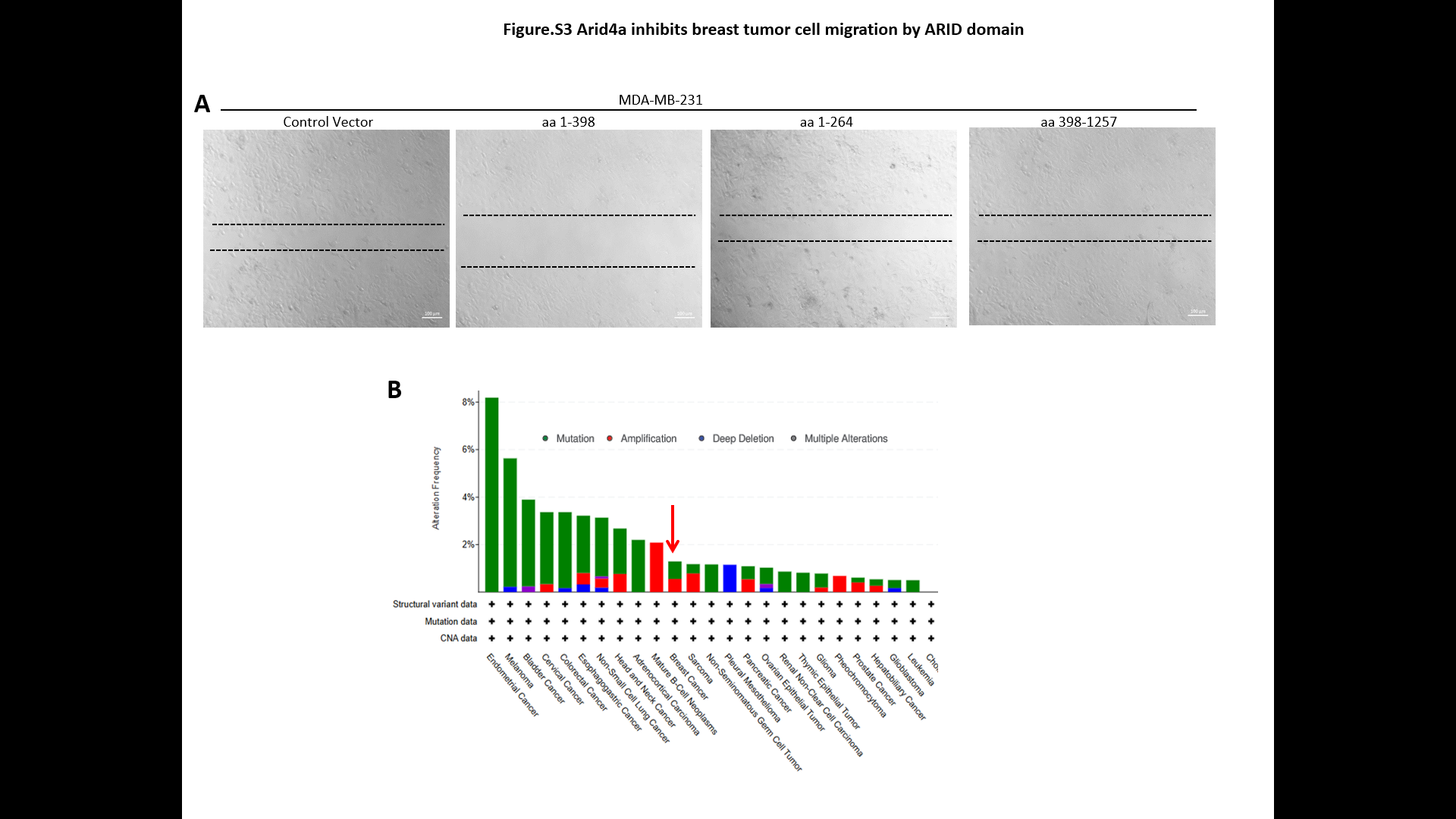
**

**Figure S4. Knockdown of Arid4a increases cell growth of breast tumor cells.** (A) Cell counting was conducted to measure the proliferation of MDA-MB-231 and MCF7 cells after the knockdown of Arid4a. (B) MTT assay was performed to measure the cell activity of breast tumor cells silencing Arid4a expression. (C) qPCR was performed to detect the mRNA expression of indicated metastasis-promoting genes in breast tumor cells after Arid4a knockdown. (D) Half-lives of the indicated mRNAs were measured after Arid4a knockdown in MDA-MB-231 cells. **P*<0.05.

**
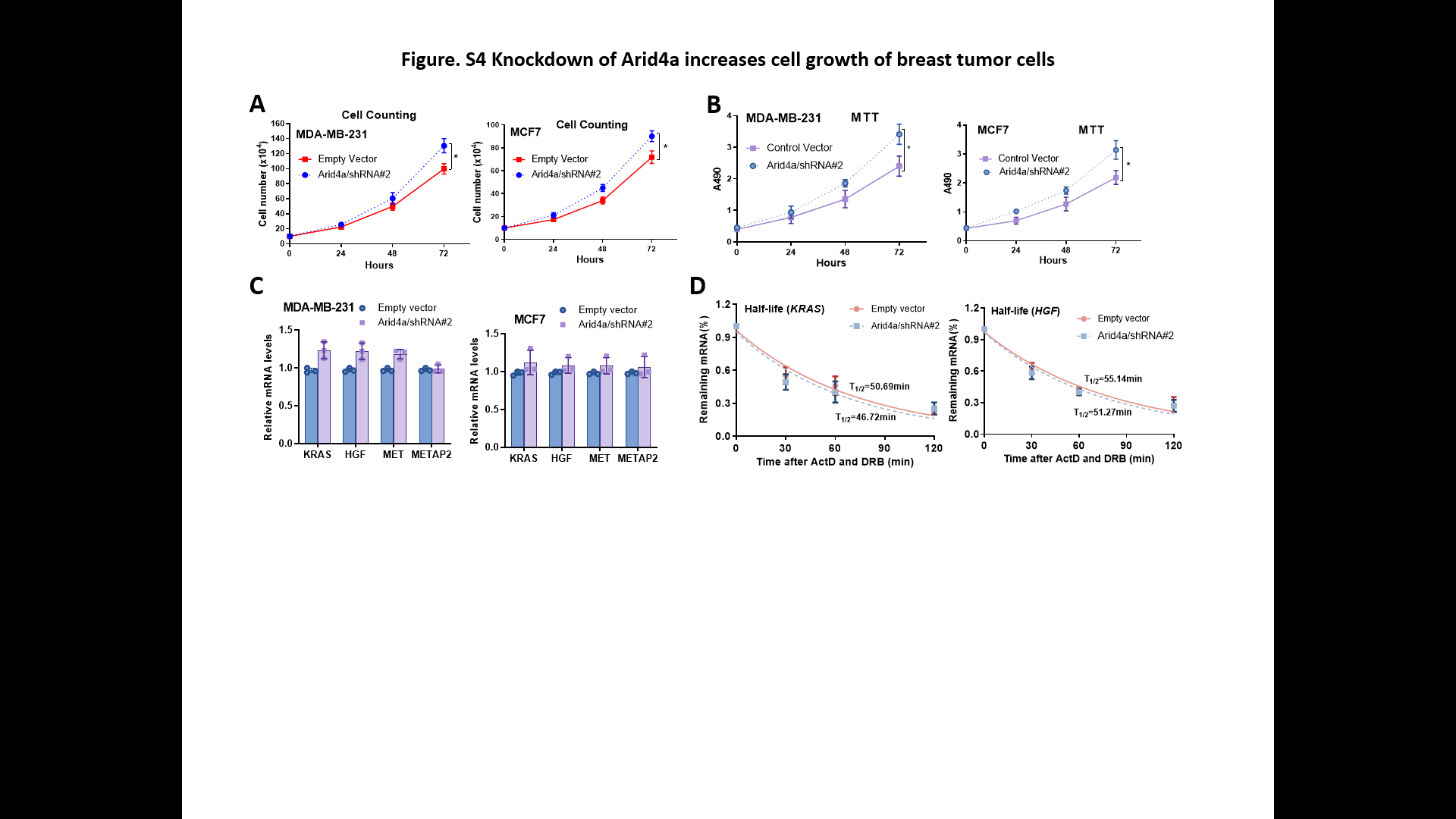
**

**Figure S5. Arid4a expression was positively correlated with MTSS1 expression in human breast cancer tissues. (**A) Comparison of MTSS1 protein expression between normal breast tissues and primary breast tumors (<http://ualcan.path.uab.edu/analysis-prot.html>). (B) Protein expression of MTSS1 in different subtypes of breast cancers (<http://ualcan.path.uab.edu/analysis-prot.html>). (C) Protein expression of Arid4a were analyzed by the main pathological stages of breast cancer (<http://ualcan.path.uab.edu/analysis-prot.html>). (D) Pearson’s Correlation between *Arid4a* and *TIMP2, RB1,* and *PTEN* expression was analyzed by TIMER2.0 ([TIMER2.0 (cistrome.org)](http://timer.cistrome.org/)) (upper) and GEPIA2 (<http://gepia2.cancer-pku.cn/#analysis>) (down), respectively, in human breast cancer patients.

**
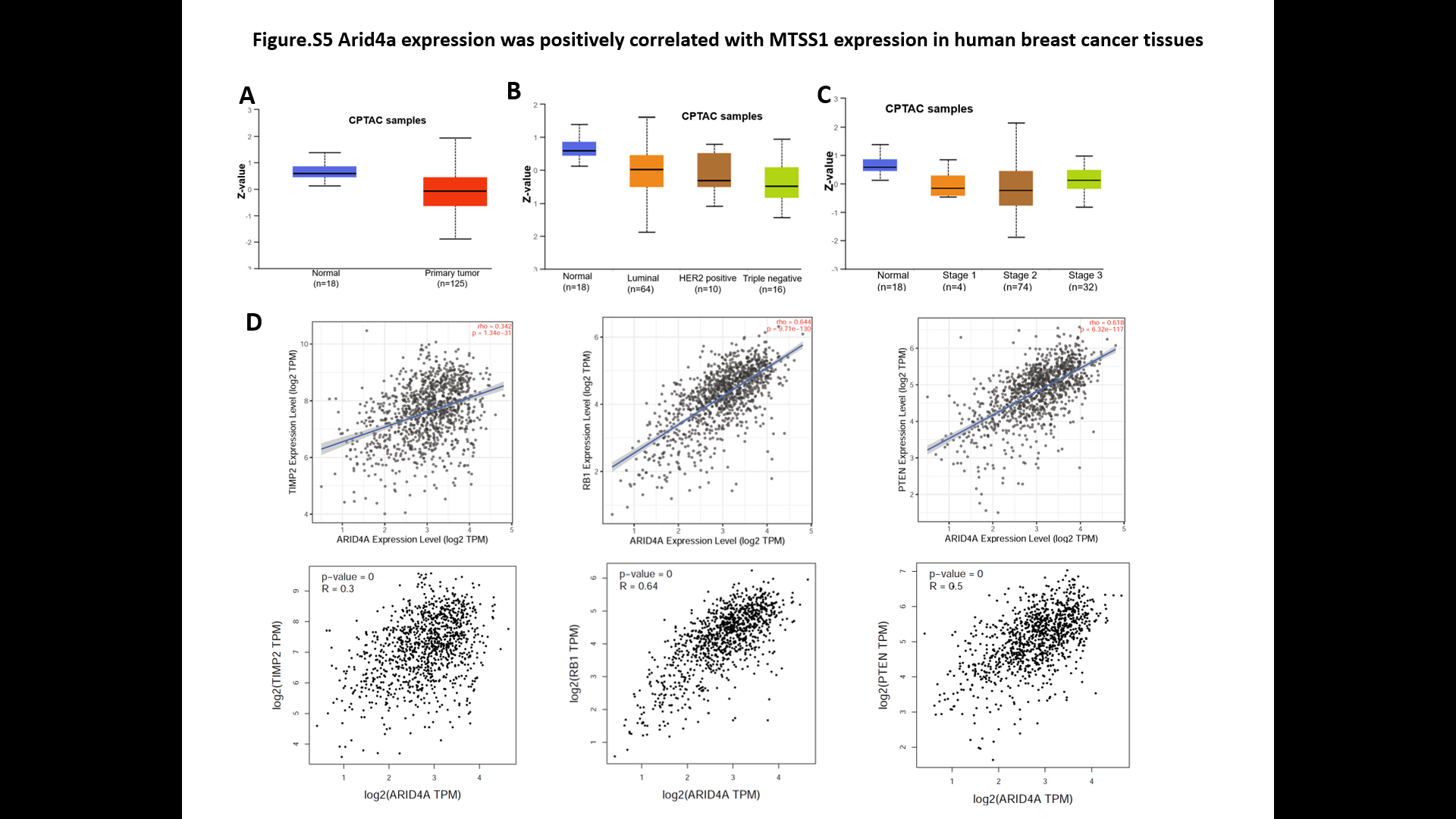
**
