# Supplementary material for: Arid4a Suppresses Breast Tumor Metastasis by Enhancing MTSS1 Expression via mRNA Stability
Source: Cancer Med. 2025 Mar 11;14(5):e70732. doi: 10.1002/cam4.70732 (PMC11894439; doi:10.1002/cam4.70732)
Supplement: Supplementary file 2 — Table S1 PCR Array analysis of human metastasis‐related genes regulated by Arid4a. [file CAM4-14-e70732-s002.docx]

**Table S1. PCRarray analysis of human metastasis-related genes regulated by Arid4a**

| **Gene Symbol** | **Gene Name** | **Control** | **Arid4a-Overexpression** | **fold change** |
| --- | --- | --- | --- | --- |
| APC | Adenomatous polyposis coli | **29.78** | **29.98** | 0.16 |
| BRMS1 | Breast cancer metastasis suppressor 1 | **29.86** | **29.81** | 0.35 |
| CCL7 | Chemokine (C-C motif) ligand 7 | **-** | **-** | NA |
| CD44 | CD44 molecule (Indian blood group) | **22.42** | **23.28** | -0.27 |
| CD82 | CD82 molecule | **24.54** | **24.29** | 0.42 |
| CDH1 | Cadherin 1, type 1, E-cadherin (epithelial) | **31.28** | **30.97** | 0.47 |
| CDH11 | Cadherin 11, type 2, OB-cadherin (osteoblast) | **-** | **-** | NA |
| CDH6 | Cadherin 6, type 2, K-cadherin (fetal kidney) | **-** | **-** | NA |
| CDKN2A | Cyclin-dependent kinase inhibitor 2A (melanoma, p16, inhibits CDK4) | **28.69** | **29.22** | -0.08 |
| CDH4 | Chromodomain helicase DNA binding protein 4 | **36.65** | **36.33** | 0.66 |
| COL4A2 | Collagen, type IV, alpha 2 | **30.25** | **29.99** | 0.59 |
| CST7 | Cystatin F (leukocystatin) | **35.64** | **35.38** | 0.41 |
| CTBP1 | C-terminal binding protein 1 | **30.32** | **30.96** | -0.15 |
| CTNNA1 | Catenin (cadherin-associated protein), alpha 1, 102kDa | **24.67** | **24.86** | 0.16 |
| CTSK | Cathepsin K | **29.45** | **29.71** | 0.10 |
| CTSL1 | Cathepsin L1 | **24.32** | **24.43** | 0.23 |
| CXCL12 | Chemokine (C-X-C motif) ligand 12 | **-** | **-** | NA |
| CXCR2 | Chemokine (C-X-C motif) receptor 2 | **-** | **-** | NA |
| CXCR4 | Chemokine (C-X-C motif) receptor 4 | **34.32** | **35.97** | -0.58 |
| DENR | Density-regulated protein | **26.67** | **26.92** | 0.12 |
| EPHB2 | EPH receptor B2 | **27.36** | **27.38** | 0.31 |
| ETV4 | Ets variant 4 | **28.16** | **28.25** | 0.25 |
| EWSR1 | Ewing sarcoma breakpoint region 1 | **25.22** | **25.12** | 0.42 |
| FAT1 | FAT tumor suppressor homolog 1 (Drosophila) | **27.28** | **27.97** | -0.18 |
| FGFR4 | Fibroblast growth factor receptor 4 | **28.57** | **29.62** | -0.36 |
| FLT4 | Fms-related tyrosine kinase 4 | **-** | **-** | NA |
| FN1 | Fibronectin 1 | **30.86** | **31.76** | -0.29 |
| FXYD5 | FXYD domain containing ion transport regulator 5 | **25.43** | **25.36** | 0.39 |
| GNRH1 | Gonadotropin-releasing hormone 1 (luteinizing-releasing hormone) | **27.18** | **28.13** | -0.32 |
| HGF | Hepatocyte growth factor | **32.46** | **34.76** | -0.73 |
| HPSE | Heparanase | **29.78** | **29.81** | 0.30 |
| HRAS | V-Ha-ras Harvey rat sarcoma viral oncogene homolog | **27.89** | **27.91** | 0.31 |
| HTATIP2 | HIV-1 Tat interactive protein 2, 30kDa | **34.30** | **34.46** | 0.19 |
| IGF1 | Insulin-like growth factor 1 (somatomedin C) | **-** | **-** | NA |
| IL18 | Interleukin 18 | **28.78** | **31.21** | -0.75 |
| IL1B | Interleukin 1, beta | **39.46** | **39.98** | -0.07 |
| ITGA7 | Integrin, alpha 7 | **30.26** | **30.01** | 0.58 |
| ITGB3 | Integrin, beta 3 | **29.87** | **31.23** | -0.49 |
| KISS1 | KiSS-1 metastasis-suppressor | **35.36** | **36.48** | -0.39 |
| KISS1R | KISS1 receptor | **-** | **-** | NA |
| KRAS | V-Ki-ras2 Kirsten rat sarcoma viral oncogene homolog | **26.61** | **30.83** | -0.93 |
| MCAM | Melanoma cell adhesion molecule | **28.67** | **29.21** | -0.09 |
| MDM2 | Mdm2 p53 binding protein homolog (mouse) | **26.36** | **27.13** | -0.22 |
| MET | Met proto-oncogene (hepatocyte growth factor receptor) | **27.27** | **30.22** | -0.83 |
| METAP2 | Methionyl aminopeptidase 2 | **27.56** | **29.69** | -0.70 |
| MGAT5 | Mannosyl (alpha-1,6-)-glycoprotein beta-1,6-N-acetyl-glucosaminyltransferase | **31.19** | **31.24** | 0.28 |
| MMP10 | Matrix metallopeptidase 10 (stromelysin 2) | **-** | **-** | NA |
| MMP11 | Matrix metallopeptidase 11 (stromelysin 3) | **37.91** | **37.82** | 0.41 |
| MMP13 | Matrix metallopeptidase 13 (collagenase 3) | **-** | **-** |  |
| MMP2 | Matrix metallopeptidase 2 | **36.56** | **36.22** | 0.68 |
| MMP3 | Matrix metallopeptidase 3 (stromelysin 1, progelatinase) | **-** | **-** | NA |
| MMP7 | Matrix metallopeptidase 7 (matrilysin, uterine) | **32.23** | **34.18** | -0.66 |
| MMP9 | Matrix metallopeptidase 9 (gelatinase B, 92kDa gelatinase, 92kDa type IV  collagenase) | **-** | **-** | NA |
| MTA1 | Metastasis associated 1 | **31.12** | **31.98** | -0.27 |
| MTSS1 | Metastasis suppressor 1 | **29.19** | **28.63** | 0.96 |
| MYC | V-myc myelocytomatosis viral oncogene homolog (avian) | **27.27** | **27.96** | -0.18 |
| MYCL1 | V-myc myelocytomatosis viral oncogene homolog 1, lung carcinoma derived  (avian) | **25.74** | **25.28** | 0.38 |
| NF2 | Neurofibromin 2 (merlin) | **26.76** | **26.79** | 0.30 |
| NME1 | Non-metastatic cells 1, protein (NM23A) expressed in | **31.12** | **31.11** | 0.34 |
| NME4 | Non-metastatic cells 4, protein expressed in | **31.33** | **31.29** | 0.36 |
| NR4A3 | Nuclear receptor subfamily 4, group A, member 3 | **29.59** | **30.13** | -0.09 |
| PLAUR | Plasminogen activator, urokinase receptor | **29.83** | **29.92** | 0.25 |
| PNN | Pinin, desmosome associated protein | **27.65** | **27.69** | 0.29 |
| PTEN | Phosphatase and tensin homolog | **28.68** | **28.29** | 0.74 |
| RB1 | Retinoblastoma 1 | **33.43** | **33.01** | 0.78 |
| RORB | RAR-related orphan receptor B | **37.35** | **37.97** | -0.14 |
| RPSA | Ribosomal protein SA | **30.28** | **31.51** | -0.43 |
| SERPINE1 | Serpin peptidase inhibitor, clade E (nexin, plasminogen activator inhibitor type 1), member 1 | **34.59** | **34.13** | 0.83 |
| SET | SET nuclear oncogene | **27.32** | **28.39** | -0.37 |
| SMAD2 | SMAD family member 2 | **28.84** | **29.73** | -0.27 |
| SMAD4 | SMAD family member 4 | **26.57** | **26.48** | 0.35 |
| SRC | V-src sarcoma (Schmidt-Ruppin A-2) viral oncogene homolog (avian) | **29.45** | **30.22** | -0.22 |
| SSTR2 | Somatostatin receptor 2 | **25.55** | **25.34** | 0.54 |
| SYK | Spleen tyrosine kinase | **29.92** | **30.84** | -0.30 |
| TCF20 | Transcription factor 20 (AR1) | **31.25** | **31.26** | 0.32 |
| TGFB1 | Transforming growth factor, beta 1 | **28.33** | **28.11** | 0.55 |
| TIMP2 | TIMP metallopeptidase inhibitor 2 | **27.33** | **26.82** | 0.89 |
| TIMP3 | TIMP metallopeptidase inhibitor 3 | **29.39** | **29.11** | 0.61 |
| TIMP4 | TIMP metallopeptidase inhibitor 4 | **38.84** | **38.66** | 0.51 |
| TNFSF10 | Tumor necrosis factor (ligand) superfamily, member 10 | **31.29** | **31.22** | 0.39 |
| TP53 | Tumor protein p53 | **27.87** | **27.43** | 0.12 |
| TRPM1 | Transient receptor potential cation channel, subfamily M, member 1 | **38.54** | **38.88** | 0.05 |
| TSHR | Thyroid stimulating hormone receptor | **39.42** | **39.70** | 0.09 |
| VEGFA | Vascular endothelial growth factor A | **27.36** | **28.89** | -0.54 |
| ACTB | Actin, beta | **21.22** | **22.41** |  |
| B2M | Beta-2-microglobulin | **24.12** | **23.35** |  |
| GAPDH | Glyceraldehyde-3-phosphate dehydrogenase | **19.13** | **19.54** |  |
| HPRT1 | Hypoxanthine phosphoribosyltransferase 1 | **28.24** | **28.73** |  |
| RPLP0 | Ribosomal protein, large, P0 | **18.58** | **18.44** |  |
| HGDC | Human Genomic DNA Contamination |  |  |  |
| RTC | Reverse Transcription Control |  |  |  |
| RTC | Reverse Transcription Control |  |  |  |
| RTC | Reverse Transcription Control |  |  |  |
| PPC | Positive PCR Control | **21.33** | **21.42** |  |
| PPC | Positive PCR Control | **21.36** | **21.69** |  |
| PPC | Positive PCR Control | **21.41** | **21.88** |  |
