# Supplementary material for: Arid4a Suppresses Breast Tumor Metastasis by Enhancing MTSS1 Expression via mRNA Stability
Source: Cancer Med. 2025 Mar 11;14(5):e70732. doi: 10.1002/cam4.70732 (PMC11894439; doi:10.1002/cam4.70732)
Supplement: Supplementary file 3 — Table S2 PCR Primers and RNA‐EMSA Probes Sequence. [file CAM4-14-e70732-s003.docx]

**Table S2. PCR Primers and RNA-EMSA Probes Sequence**

| Gene name | Primer (sense) (5’-3’) | Primer (anti-sense) (5’-3’) |
| --- | --- | --- |
| *huArid4a* (qPCR) | GAGGTGCCTTCTGTGAGGCAAAGATTA | TACGTCTCAATGTTCGCTCATCACCAT |
| *huGAPDH* (qPCR) | GCTTGTCATCAATGGAAATCCCATCAC | TGATCTTGAGGCTGTTGTCATACTTCTC |
| *huPTEN* (qPCR) | GACACCGCCAAATTTAATTGCAGAGTTG | TCCTTACTTCCCCATAGAAATCTAGGGC |
| *huRB1* (qPCR) | TGGGGAATCTGTATCTTTATTGCAGCA GT | AGTAGATATCGAACTGCTGGGTTGTGTC |
| *HuTIMP2* (qPCR) | AACAGGCGTTTTGCAATGCAGATGTAG | CCTCGGCCTTTCCTGCAATGAGATATT |
| *huMTSS1* (qPCR) | GCAGAAGAAAGCAAAAAAAGGGAGAGGT | TGGTCAGGCTTTTTAGATCTTCCGAGAT |
| *hu* *METAP2* (qPCR) | AGATGAAGATGATGAAGATGGAGATGGCG | CCAAATCTCTTCACTTGCCTGATCTAATGC |
| *hu* *MET* (qPCR) | CCTTCAGAAGGTTGCTGAGTACAAGACT | AGAATATGCAGTGAACCTCCGACTGTAT |
| *hu* *KRAS* (qPCR) | TGTCTCTTGGATATTCTCGACACAGCAG | CTTCTTGCTAAGTCCTGAGCCTGTTTTG |
| *hu* *HGF* (qPCR) | TCTGGTTCCCCTTCAATAGCATGTCAA | CCTCGAGGATTTCGACAGTAGTTTTCCT |
| *huPTEN* (full-length 3’UTR) | TTCTAGAATCAAGAGGGATAAAACACCATGAAAATAA | GGCCGGCCATACTCTCCATAGGAAGGCATTTCTATTT |
| *huTIMP2* (full-length 3’UTR) | TTCTAGAAACTGCAAAAAAAGCCTCCAAGGGTTTCGA | GGCCGGCCCTCTTTGATAATACT GTTTATTGTCCACGG |
| *huRB1* (full-length 3’UTR) | TTCTAGAGGATCTCAGGACCTTGGTGGACACTGTGTA | GGCCGGCCTTAAGACTCTTAACAACTAATTTATTTTAA |
| *huMTSS1* (full-length 3’UTR) | TTCTAGATTCACAAGAAATGCGCCGGTGGGGAATGAA | GGCCGGCCTAACGTAGGCTGAAGCAATTGTTACAATGTA |
| *huMTSS1* (truncated 3’UTR) | TTCTAGATTCACAAGAAATGCGCCGGTGGGGAATGAA | GGCCGGCCTTGGTTTTAATTCTTATCTAAAGGTGTCGA |
| *huMTSS1* (3’UTR-mut1) | CTGCCCAATGCCUAAAUAACUGACAUAAU | CAAAGGGGTAGGATGTGAACCAGTATATCACAAAGCT |
| *huMTSS1* (3’UTR-mut2) | CTGCCCAATGCCTAGATAACTGATCTAAT | CAAAGGGGTAGGATGTGAACCAGTATATCACAAAGCT |
| *huMTSS1* (RNA-ChIP) | CGGTGGGGAATGAACTGTTTCATTAATAA | AGTTAAATGGAATTTGGCACCTTCAGAAA |
|  |  |  |
| *MTSS1* (WT-Probe): 5’- CCUAUGUAACUGACAUAAU -3’  *MTSS1* (mut-Probe): 5’- CCUAAAUAACUGACAUAAU -3’ | | |
