# Supplementary material for: Arid4a Suppresses Breast Tumor Metastasis by Enhancing MTSS1 Expression via mRNA Stability
Source: Cancer Med. 2025 Mar 11;14(5):e70732. doi: 10.1002/cam4.70732 (PMC11894439; doi:10.1002/cam4.70732)
Supplement: Supplementary file 4 — Data S1 [file CAM4-14-e70732-s001.docx]

**Supplementary Material S1**

**Antibodies and Reagents**

Polyclonal rabbit anti-Arid4a (A302-231A) was from Bethyl Laboratories. Polyclonal rabbit anti-GFP (ab290), and normal rabbit IgG (ab37415) were from Abcam. Polyclonal rabbit beta-actin (20536-1-AP) and MTSS1 (13995-1-AP) antibodies were from Proteintech. Thiazolyl Blue Tetrazolium Bromide (MTT) (M2128), G418 (G8168), puromycin (P8833), actinomycin D (ActD, A1410), and 5, 6-Dichlorobenzimidazole 1-β-D-ribofuranoside (DRB, D1916) were from Sigma. Protein A/G PLUS-Agarose beads (sc-2003) were from Santa Cruz Biotechnology. GFP-coated beads (gta-20) were from ChromoTek (Germany).

**Plasmids**

The human full-length Arid4a coding sequence (NM_002892.3) was synthesized, sequenced and inserted into pEGFP-N1 vector at Nhe I and Hind III sites. Arid4a serial deletion plasmids were generated by inserting the PCR-amplified fragments into pEGFP-N1 vector at EcoR I and Age I sites. A set of luciferase reporters were constructed by inserting the full-length 3'UTRs of human *MTSS1*, *TIMP2*, *RB1*, and *PTEN* into the pGL3 control vector (Promega) between Xba I and Fse I sites, respectively. For stem–loop deletion reporters, point mutated and truncated *MTSS1*-3'UTR (∆stem–loop) were amplified, sequenced, and inserted into pGL3 control vector using Phusion Site-Directed Mutagenesis Kit (Thermo Scientific).

**qRT-PCR**

**Western Blotting**

Cell or tissue samples were collected and lysed with RIPA buffer containing PMSF and protease inhibitor cocktail (Roche, Switzerland). Protein concentration was measured by BCA method. Equal amount of protein lysates was subjected to electrophoresis by SDS-PAGE and transferred onto a polyvinylidene membrane. The membrane was then blocked with 5% fat-free milk and incubated with primary antibody (1:1000, diluted in 5% fat-free milk) overnight at 4℃. The band was detected with HRP-conjugated secondary antibodies (1:3000; Santa Cruz) using ECL chemiluminescent detection method.

**Cell cycle analysis**

Cells were harvested and washed twice with PBS, and then fixed in cold ethanol (70%). The cells were then stained with propidium iodide (20 µg/mL) and RNase A (0.2 mg/mL) for 30 min. The stained cells (at least 1 x 10^4^ cells) were analyzed by flow cytometry and the data were analyzed with FlowJo software. All cell cycle analysis was performed in triplicate.

**Immunohistochemistry (IHC) and hematoxylin & eosin (H&E) staining**

Tumor tissues were subjected to IHC to detect the expression of interested proteins using indicated antibodies. Mouse lung tissues have been immersed in 10% formalin to soak for at least two weeks and then treated with H&E for staining. Zeiss Imaging System was used to visualize the staining sections.

**Cell counting assay**

Breast tumor cells were seeded at the total number of 10 x 10^4^ cells/well in a 6-well plate and cultured with DMEM or RPMI-1640 with 10% FBS plus 1% Peni/Stro. Cells were counted at 24, 48, and 72 hrs. Each cell line was plated in triplicate and the experiment was repeated at least three times with consistency.

**MTT assay**

1 x 10^4^ cells/well were cultured in 96-well plate with DMEM or RPMI-1640 with 10% FBS plus 1% Peni/Stro. A total of 20μL of MTT dilution in PBS (5 mg/ml) was added per well, and then cells were incubated for 4 h. After adding DMSO and shaking for 10 mins, absorbance was measured at 490 nm using a Bio-Tek Elx800 Series Universal Microplate Reader.

**RNA immunoprecipitation (RIP)**

Total protein extracts were obtained from MDA-MB-231 or MDA-MB-231/Arid4a-GFP cells and precleared with IgG, followed by incubation with anti-Arid4a, anti-GFP antibodies or isotype IgG at 4℃ for 4 hours, respectively. RNA-protein complexes were immunoprecipitated with protein A/G agarose beads or GFP-coated beads (gta-20). The pulled down RNAs were extracted from the beads and reverse transcribed to cDNA for qPCR or RT-PCR detection.

**Luciferase reporter assays**

Expressing Arid4a/GFP, GFP-control constructs and pGL3 luciferase reporter constructs containing full-length or mutant of 3'UTR of MTSS1 gene were cotransfected into HEK293 cells using Lipofectamine 2000 reagent, respectively. After 48 h, the cells were lysed with 1 × reporter lysis buffer and luciferase activities were measured using a Dual-Luciferase Reporter Assay System (Promega, USA). All experiments were conducted in triplicate and repeated at least three times.

**shRNA lentivirus**

Two lentiviral shRNAs (TRCN0000014595; TRCN0000014596) targeting the CDS of human Arid4a mRNA were purchased from Merck. A scramble control shRNA was used as a control. Lentiviral particles were packaged in HEK293T cells by cotransfecting shRNA-pLKO.1, pCMV-dR8.2, and pMD2.G constructs. After infection, the target cells were selected with puromycin (1.0µg/mL), followed by further study.

**Study approval**

All subjects gave their informed consent for inclusion before they participated in the study. The study was conducted in accordance with the Declaration of Helsinki, and approved by the Medical Ethics Committee of the Institute of Microcirculation, CAMS & PUMC.

**RNA-EMSA**

20 fmol of the 3'-end biotin-labeled WT and mutant probes were incubated with 15 μg of total cell lysates containing Arid4a/GFP fusion protein for 30 min at room temperature with 20 μl of binding buffer containing 10 mM Tris, 50 mM KCl, 1 mM Dithiothreitol (DTT) (pH 7.5). RNA-protein complexes were resolved by 6% non-denaturing polyacrylamide gel electrophoresis, and the gel was transferred onto a nylon membrane and UV-cross linked. The membrane was then subjected to detection by chemiluminescent EMSA kit (Pierce) following the manufacturer’s protocol.

**RNA-ChIP**

Arid4a/GFP fusion protein was expressed in MDA-MB-231 cells. Then, cells were cross-linked for 10 min by addition of formaldehyde (1% v/v). Glycine was used to stop crosslinking (125 mM). Cells were washed with cold PBS, resuspended in 500 μL of polysome lysis buffer, and placed on ice for 5 min. Cell lysates were collected by centrifugation at 10,000 g for 10 min at 4°C, and re-suspended in 500 μL of polysome lysis buffer. The lysates were sonicated and pre-clear with rabbit IgG to remove non-specific background. Pre-cleared lysates were used for IP with GFP-coated beads or rabbit IgG-coated beads at 4 °C for 4 h. After pull-down, 100 μL supernatants were taken out for Input. Each immune complex was washed five times with ice-cold NT2 buffer. RNA was isolated with Trizol reagent, and re-suspended in 50 μL of RNase-free water, followed by DNase I treatment and further detection.

**Sequence Alignments and Stem–loop Structure Prediction**

For 3'UTR stem–loop structure sequence conservation analysis of *MTSS1*, the 3'UTR sequences were extracted for different species from the National Center for Biotechnology Information (NCBI) database: human (*Homo sapiens*; accession number NM_014751.6), chimpanzee (*Pan troglodytes*; XM_016959836.2), mouse (*Mus musculus*; NM_144800.3), rat (*Rattus*; NM_001130563.1). Stem–loop sequence conservation analysis was performed using DNAMAN software. The stem–loop structure was predicted through RNAfold web server ( <http://rna.tbi.univie.ac.at/> ).
